# Adaption and pilot testing of a lay HIV supporter program for traditional healers: a mixed methods study in rural Uganda

**DOI:** 10.1186/s43058-023-00469-5

**Published:** 2023-07-27

**Authors:** Radhika Sundararajan, Matthew Ponticiello, Giselle Birch, Gabriel Nuwagaba, Rinu Alaiku, Denis Nansera, Juliet Mwanga-Amumpaire, Winnie Muyindike

**Affiliations:** 1 Center for Global Health, Weill Cornell Medicine, New York, NY USA; 2 Department of Emergency Medicine, Weill Cornell Medicine, New York, NY USA; 3 Yale School of Medicine, Yale University, New Haven, USA; 4 University College London, London, UK; 5 Mbarara University of Science and Technology, Mbarara, Uganda; 6 Weill Cornell Medical College, Weill Cornell Medicine, New York, USA; 7 Mbarara Regional Referral Hospital, Mbarara, Uganda

**Keywords:** ADAPT-ITT, Intervention adaptation, HIV care, Lay support, Africa

## Abstract

**Background:**

Half of people living with HIV (PLWH) in sub-Saharan Africa default from care within two years. In Uganda, and across sub-Saharan Africa, traditional healers (TH) are ubiquitous and often serve as the first line of health care. We hypothesized that with lay support training, TH could support relinkage to HIV care and ART adherence among rural Ugandan PLWH who have defaulted from HIV care.

**Methods:**

Following the ADAPT-ITT framework, we adapted an evidence-based layperson HIV support program from South Africa for delivery by Ugandan TH. The ADAPT-ITT framework consists of (1) *A*ssessment of needs; (2) *D*eciding which evidence-based interventions to adapt; (3) *A*daptation of interventions; (4) *P*roduction of drafted adapted interventions; (5) *T*opical expert feedback; (6) *I*ntegration of expert feedback; (7) *T*raining personnel; and (8) *T*esting the adapted intervention. The *Testing* phase was completed via a pilot mixed methods prospective cohort study. The study population included 12 TH practicing in Mbarara Township and 20 adult PLWH with suboptimal ART adherence (CASE adherence index score < 10) who received care from a participating TH and who resided in Mbarara Township. Primary outcome was re-linkage to HIV care within 14 days. Secondary outcomes were ART re-initiation, ART adherence, retention in care after 9 months, and implementation measures. Qualitative interviews were conducted with all participants.

**Results:**

Data from the *Assessment* phase indicated that logistical challenges played an important role in disengagement from care among PLWH who receive care from TH, notably geographical distance to clinics and transportation costs. Additionally, HIV-related stigma and lack of social support were identified as barriers to entering and remaining in HIV care. Two core elements of the intervention were identified during the *Production* phase: (1) TH facilitating rapid re-linkage to HIV care and (2) TH provision of psychosocial support. In the pilot study phase, baseline median CASE adherence score was 3; only 5% of PLWH were adherent to ART via 4-day recall. The TH-delivered support achieved 100% linkage and ART initiation within 14 days, 95% ART adherence, and 100% of PLWH were retained in HIV care after 9 months.

**Conclusions:**

The ADAPT-ITT framework successfully guided the adaption of a community health worker-delivered intervention for delivery by TH. TH successfully facilitated re-linkage to HIV care, support ART adherence, and retention in care for PLWH when trained as part of a lay support person program. Future studies are needed to evaluate scale-up and long-term impact.

Contributions to the literature

- We describe the adaption process of an intervention for people living with HIV (PLWH) who have defaulted from care following the ADAPT-ITT framework
- Interventions developed for community health workers may be well suited for adaption for delivery by traditional healers
- Accounting for context-specific logistical barriers and cultural preferences during program adaptation can maximize intervention feasibility, appropriateness, and acceptability, ultimately improving clinical outcomes for patients
- Traditional healers should be considered as stakeholders in implementation and scale-up of community-based interventions

## Introduction

People living with HIV (PLWH) who reside in rural areas have disproportionally worse HIV outcomes than urban PLWH in sub-Saharan Africa. In Uganda, for example, HIV prevalence in rural areas is as high as 17%, compared to the national prevalence of 6% [1]. Moreover, only 39% of rural PLWH are on ART and 23% are virally suppressed, as compared to 75% and 59% of urban dwellers, respectively [2]. Across the HIV continuum of care, engagement of PLWH living in rural areas is 20–40% lower than their urban counterparts, falling strikingly short of the UN AIDS Program 95–95–95 benchmarks to end the epidemic [3]. Geographic distance from clinics and transportation-related barriers impede access to HIV care for rural populations, resulting in greater HIV clinic absenteeism [4, 5]. The aforementioned disparities illustrate the critical unmet need for effective strategies to improve access to and retention of PLWH along the HIV continuum of care in rural communities.

In Uganda, as across much of sub-Saharan Africa, over 80% of the population reports utilizing traditional healers (TH) [6]. TH are informal healthcare providers and trusted community members who may be uniquely positioned to bridge the rural HIV treatment gap. Qualitative data from multiple countries suggest that TH are among the most accessible and trusted providers in rural communities, where the majority of Africans reside [7–12]. TH often serve as the first line of healthcare for rural PLWH, some of whom prefer healers’ care alone, and some of whom utilize both TH and clinic-based care [13–16]. TH also provide care for rural PLWH who have disengaged from HIV care [17]. The accessibility and pre-existing community trust of TH suggest that they may be ideal lay providers to connect rural PLWH to life-saving HIV clinical care [18, 19].

There is some evidence that TH can improve the HIV continuum of care. Studies in East and South Africa suggest that TH can educate their clients on HIV prevention such as condom use, risk reduction, and HIV testing [20–23]. A pre-post non-randomized study in rural Mozambique found an educational program for TH increased biomedical referrals for adults with symptoms of HIV, TB, or malaria (35% increase, *p* = 0.046) [23]. A cross-sectional study of Zimbabwean PLWH reported a higher quality of life when using healers plus HIV clinical care, compared to clinical care alone; however, the impact on ART adherence and viral suppression was not quantified [14]. However, other cross-sectional and cohort studies suggest that African PLWH who use TH experience delayed HIV diagnosis and ART nonadherence, potentially because healers in those studies were neither aware of HIV symptoms nor integrated with HIV clinical care [15, 17, 24, 25]. We propose that with rigorous lay provider training, establishing collaborative relationships with HIV clinicians, and with consideration of implementation determinants, TH can be effective supporters to improve HIV outcomes among rural PLWH.

Here, we describe the process of adapting an evidence-based program using the ADAPT-ITT framework and present results from a pilot study to evaluate if TH-delivered lay support can improve entry and retention in HIV care for PLWH who have defaulted from care. ADAPT-ITT has been successfully applied in similar contexts to adapt evidence-based HIV programs for delivery [26–28]. We selected the PEPFAR-supported Patient Advocate Program for adaption. This community health worker-delivered intervention was developed for PLWH in South Africa and assists linkage to HIV care, supports ART initiation and adherence through medication counseling, and facilitates retention in care through the provision of individual psychosocial support. A multi-center cohort study among ~ 67,000 PLWH at 57 sites in South Africa [29] showed that the Patient Advocate Program improved viral suppression 8 years after ART initiation, compared with routine clinical care (88.6 vs 80.6%, adjusted RR = 1.53) [30]. This evidence-based program required adaptation for our setting as it was developed in a semi-urban South African context with a robust force of community health workers, unlike rural Uganda. We adapted the program for TH given that they are accessible and trusted lay providers in rural Ugandan communities where access to health facilities is more difficult, literacy is lower, and baseline viral suppression is poorer than where the original intervention was tested.

## Methods

### Study design

We conducted a mixed-methods study that included gathering stakeholder input to identify and adapt an evidence-based intervention, followed by a prospective cohort study with individual qualitative interviews. This study was conducted in Mbarara Township in southwestern Uganda. Mbarara Township is a rural, agriculture-producing region located about 270 km from the capital city of Kampala. Mbarara District’s largest government-supported HIV clinic provides free care to a catchment area of about 475,000 residents. HIV prevalence in Mbarara is 7.9%, surpassing Uganda’s national HIV prevalence of 5.8% [31]. We have been collaborating with TH since 2017 to improve HIV care in the region and have demonstrated that adults who receive care from TH have low rates of HIV testing [32] and that TH can improve uptake of HIV through the delivery of counseling and distribution of HIV self-testing at their practices [33, 34].

### Eligibility criteria

In this pilot study, we trained TH in an adapted evidence-based curriculum of lay support for PLWH who have defaulted from HIV care and are seeking traditional care at TH practices. TH were eligible to participate if they were aged 18 years or older; were identified in the 2018 population-level census of TH in Mbarara District; were located within 8 km of the Mbarara District HIV clinic; provided care for PLWH; and delivered care to at least seven clients per week. Inclusion criteria for PLWH clients were age 18 years or older; ART naïve or suboptimal ART adherence defined as CASE Adherence Index Score < 10 [35]; received care from a participating healer; and reside in Mbarara Township. Eligibility screening and informed consent took place at healer locations. All participants, including healers and their clients, were provided with written informed consent in the local language (Runyankole) by a Ugandan research assistant. In the case of participant limited literacy, the informed consent was read aloud in front of a witness and the participant provided a fingerprint in place of a signature. This study was approved by the institutional review boards of Mbarara University of Science and Technology (Mbarara, Uganda), Ugandan National Council of Science and Technology, and Weill Cornell Medicine (New York, USA).

### Screening and enrollment

Healers were provided with eligibility criteria for the trial and screened all clients receiving care at their practices during the enrollment period. If a client was determined to be potentially eligible, the healers would contact a study research assistant, who would arrive at the practice within one hour to confirm eligibility and conduct study recruitment and enrollment procedures, if the client agreed. We recruited individual clients receiving care from participating healers on a rolling basis. Screening and recruitment were continued at each healer site until target enrollment was reached. All eligible participants approached agreed to participate in the study.

### Adaptation of an evidence-based intervention

We followed the stepwise ADAPT-ITT framework to identify an evidence-based lay support program and adapt it for use among Ugandan TH. The ADAPT-ITT framework consists of (1) *A*ssessment of needs; (2) *D*eciding which evidence-based intervention to adapt; (3) *A*daptation of the intervention; (4) *P*roduction of drafted adapted interventions; (5) *T*opical expert feedback on adapted intervention draft; (6) *I*ntegration of expert feedback; (7) *T*raining personnel; and (8) *T*esting the adapted intervention. Steps of the ADAPT-ITT process are described below and summarized in Table 1.

**Table 1:** Summary of ADAPT-ITT steps undertaken to select and adapt an evidence-based lay supporter intervention for Ugandan TH

ADAPT-ITT stage	Procedure(s)	Process(es)	Date completed
*Assessment*	Determined stakeholder interests in using TH to deliver non-clinical support for PLWH in rural Uganda	• Three focus groups (12 TH and 10 PLWH) in Mbarara • Drafted adaptation of Patient Advocate Program from South Africa [27] based on qualitative data from focus groups, tailoring with language specific for Ugandan TH	June 2021
*Decision*	Selected evidence-based content from similar context to adapt for Ugandan TH		
*Administration*	Theatre test and stakeholder assessments of intervention draft version	12 TH Advisory Board, 10 HIV clinicians, and 10 PLWH watched theatre presentations then participated in focus groups, and completed implementation surveys	July 2021
*Production*	Revisions made incorporating stakeholder feedback	Identified intervention core elements and created fidelity plan	July 2021
*Topical Expert Input*	Topical experts on HIV care and traditional medicine reviewed intervention draft	Gathered comments and additional revisions	August 2021
*Integration*	Integrated expert feedback	Produced a finalized draft of the intervention materials	August 2021
*Training*	Deliver training in adapted intervention	Trained clinic staff to deliver curriculum to TH, then conducted 2-day training session with TH	August 2021
*Testing*	Pilot tested adapted lay supporter intervention	9-month evaluation of HIV and implementation outcomes	Sept 2021–May 2022

We began the *Assessment* phase, with focus group discussions with TH to understand existing barriers to HIV care experienced by their clients and determine interest in involving TH as lay supporters. Guided by input from the TH in the *Decision* phase, we completed a scoping literature review to identify evidence-based strategies used to improve HIV care in similar contexts. We reviewed literature for evidence-based layperson support interventions that improved HIV viral suppression in other resource-poor settings and determined appropriateness for adaptation based on whether the intervention’s core components addressed barriers identified during the Assessment phase. We selected the PEPFAR-supported Patient Advocate Program [29] because this community health worker-delivered intervention addressed many of the identified barriers in the Assessment phase.

Next, adapted training materials were professionally translated into Runyankole and tailored for low literacy users following guidelines for cross-cultural translation, adaption, and validation of survey instruments [36]. In the *Administration *phase, we presented this adapted program via theatre presentations to a group of stakeholders: TH, HIV clinicians, and PLWH in Mbarara. Theatre presentations summarized the adapted program and included examples of how TH would deliver lay support to a PLWH. After the presentations, all attendees were invited to participate in focus groups and complete Acceptability of Intervention Measure (AIM), Intervention Appropriateness Measure (IAM), and Feasibility of Intervention Measure (FIM) surveys, which assess intervention characteristics, with higher scores indicating favorable evaluation [37]. These scales were verbally administered to maximize comprehension among participants with limited literacy. The adapted program was modified based on feedback from these theatre presentations as part of the *Production* phase. Revised program materials were shared with topical experts on HIV care in low-resource settings and traditional medicine for comments and feedback (*Topical Expert Input*), which was then integrated to create a finalized version of the adapted lay support training curriculum and evaluation materials (*Integration*). Final curriculum for the TH-facilitated lay support program is shown in Table 2.

**Table 2:** TH training curriculum, adapted from the South African Patient Advocate program [27, 28]

**Day one**
1. HIV/AIDS overview a. Epidemiology of HIV in Uganda and HIV transmission b. Rationale for ART use and adherence (U = U)
2. Ethics of working with PLWH a. Confidentiality and status disclosure b. Discrimination and stigma
3. Overview of clinical services for PLWH a. Enrollment and pre-ART counseling b. ART initiation c. Treatment of other opportunistic infections d. Adherence support and monitoring
4. Community resources for PLWH a. NGO or government offices b. Community support groups c. Advocacy groups for stigma reduction
5. Identifying mental health symptoms a. Introduction to depression and anxiety b. Common symptoms and solutions c. When to refer PLWH for further management
**Day two**
1. ART readiness, adherence barriers/facilitators
2. The role of lay adherence supporters a. Supporting PLWH outside the clinic b. Lay support versus clinical care roles c. Lay supporter as advocate and confidante
3. ART adherence support strategies a. Strengths-based counseling/self-efficacy b. Managing ART side effects c. Emphasizing goals of treatment
4. Communication skills for counseling
5. Strategies for healthy/positive living a. Nutrition, exercise, and mental health b. Routine healthcare and clinic visits c. Safer sex
6. HIV status disclosure and social support a. Counseling on disclosure strategies b. Improving social support

In the *Training* phase, we identified 12 eligible TH practicing in the Mbarara area and invited them to participate in this study as lay supporters. We trained them in the adapted curriculum during a two-day session where clinicians, counselors, social workers, and peer supporters from the District HIV clinic led the trainings. Participating healers received training on HIV transmission, the role of ART, Ugandan Ministry of Health guidelines on linkage to care, ethical principles in working with PLWH, HIV stigma, ART readiness counseling, mental health issues, strategies for healthy living with HIV, and delivering adherence support counseling. Educational content was overseen by author DN, a Ugandan infectious disease physician and clinical director of the Mbarara District HIV clinic. Each content section concluded with individual verbal assessments of understanding and structured role playing to assess appropriate support delivery. All 12 TH were required to demonstrate ≧85% competency on assessments of understanding and appropriate delivery of support at the end of the training sessions before continuing to the next content section.

The *Testing* phase took place between September 2021 and May 2022. We enrolled PLWH receiving care from TH who attended the trainings. Participating PLWH were followed for 9 months. A registered nurse at the District HIV served as a liaison to communicate with participating TH to assist with arranging re-linkage to care appointments for PLWH.

### Pilot testing data collection

Once enrolled in the study at a TH site, PLWH were assigned a de-identified study number, which was placed on a recording form. The form was completely graphical, and reading competency was not required to complete it. Research assistants provided participating TH with the CASE Adherence Index Questionnaire forms to conduct the initial screening of participants. If PLWH clients scored less than 10 on the CASE Adherence Index, they were instructed to contact a research assistant to consent and enroll the participant. For PLWH scoring > 10 on the CASE Adherence Index, TH were asked to provide general counseling on the importance of ART adherence and information on nearby HIV clinics where they could receive care.

Following enrollment, TH would contact the designated HIV clinic liaison to schedule the initial linkage to care appointment for the identified PLWH. PLWH were then given a transportation stipend equivalent to $5 USD to cover the cost of reaching the clinic for their initial visit and were given the option for the healer to accompany them to their appointment at their discretion. Following the initial linkage to care, healers delivered psychosocial support weekly for a month, then monthly for a total of 9 months. Ugandan HIV clinics provide roundtrip transportation remuneration for patients to attend subsequent appointments. TH received compensation for time spent on study-related procedures, including screening for eligible clients, counseling participating PLWH, and in some cases accompanying PLWH clients to initial clinical appointments. This stipend was roughly equivalent to $20 USD per month.

PLWH were informed during enrollment that they would be contacted after 14 days by telephone to assess for linkage to care and ART initiation and again monthly for the duration of the study period to assess retention in care. All TH, PLWH, and the HIV clinic liaison included in the trial were invited to participate in the two qualitative interviews after study completion — the first after 9 months, and the second after 12 months. The goal of the interviews was to explore the future potential of including TH in subsequent steps of the HIV continuum of care (i.e., prospective implementation needs, barriers, and facilitators), and the potential sustainability of this approach. Interviews were conducted in person by trained Ugandan research assistants. TH, PLWH, and clinical liaison interviews contained similar questions, asking about their experiences participating in the study and factors relevant to the study’s primary and secondary outcomes. Interviews lasted approximately 60 min, were conducted in the local language (Runyankole) in a private setting of the participant’s choice, and were audio recorded. Interviews were transcribed and translated into English by the interviewing research assistants, who are fluent in both Runyankole and English. Research Assistants were trained in qualitative data collection methods and had significant experience conducting qualitative research with TH and PLWH since 2018. Audio files were maintained in a secure, encrypted OneDrive file.

### Outcomes

Outcomes were assessed among individual PLWH of participating TH. The primary outcome of the pilot study was re-linkage to HIV care within 14 days. The primary outcome was assessed via self-report at the time of 14-day follow-up phone call and confirmed via the HIV clinic, with the participants’ consent. Secondary outcomes were ART (re)initiation, ART adherence, retention in care at 9 months, and program implementation outcomes (acceptability, feasibility, and appropriateness). ART adherence was assessed via self-report CASE Adherence Index Score and 4-day recall at the time of follow-up phone calls. Retention in care at 9 months and implementation outcomes were assessed via self-report at the time of 9-month follow-up. Outcomes were defined before pilot initiation and were not altered after study commencement.

### Data analysis

As the goal of this pilot study was to assess a novel intervention on a small scale in the interest of future scale-up, it was not statistically powered. Sample size of 12 TH and 20 PLWH was determined by the feasibility of completing the *Testing* phase within a 9-month period. Participant characteristics and quantitative study outcomes were summarized using descriptive statistics. English transcripts were reviewed by authors RS, MP, RA, and GB, and analyzed following a content analysis approach [38] with the intent to identify themes relevant to the study’s clinical outcomes and to explore participant experiences in the study. Illustrative quotes were selected to demonstrate themes in the interview data.

## Results

This study was conducted from June 2021-August 2022. Characteristics of TH and PLWH study participants are shown in Table 3. Qualitative results from stages of the adaptation process are identified by number in the text below, with illustrative quotes shown in Table 4.

**Table 3:** Characteristics of the Testing phase participants

**Characteristic**	*N* (%) or median [IQR]	
	*Traditional Healers (n* = *12)*	*PLWH (n* = *20)*
Age (in years)	43 [36-57]	36 [25-47]
Male gender	7 (58%)	9 (45%)
Married	10 (83%)	5 (25%)
Occupation	Spiritual healer 5 (42%) Herbalist 4 (33%) Birth attendant 2 (17%) Bonesetter 1 (8%)	Laborer 15 (75%) Business owner 5 (25%)
Christian religion	11 (92%)	19 (95%)
Years since HIV diagnosis	n/a	7 [2 - 18]
Days since the last clinic visit	n/a	83.5 [30 - 230]
Days since the last ART use	n/a	57.5 [4 - 449]^a^
Baseline CASE adherence	n/a	3 [1-5]^b^
100% ART adherence via 4-day recall [21]	n/a	1 (5%)
ART naïve	n/a	4 (20%)

^a^Excludes those who are ART naïve

^b^CASE index is out of a possible 16 points

**Table 4:** Summary of qualitative results from interviews

Qualitative findings	Theme	Illustrative quote
Factors influencing disengagement from HIV care	Logistical challenges	Quote 1: “Transport was always a challenge because of poverty and yet the distance to the HIV clinic was too long to walk. That was another de-motivating factor and discouraging [for PLWH].” – 69-year-old male, traditional healer
	Stigma	Quote 2: “Some of these people were stigmatized to the extent that whenever they tempted to go to the HIV clinic, they would feel out of place, become uncomfortable and end up getting more stressed instead. Sometimes they would just go back home without a single treatment. Some of them actually gave up completely [on clinical care].” – 36-year-old female, traditional healer
	Lack of social support	Quote 3: “I did not have any hope. I did not have anyone to support me, no one to lean on, no motivation. I had reached an extent of giving up my HIV treatment.” – 40-year-old female, PLWH
	Lack of trusting relationships with HIV clinical staff	Quote 4: “[My client living with HIV] has had many challenges with the HIV clinics, and she fails to open up and disclose her adherence issues to them” 42-year-old female, traditional healer
TH could overcome barriers to HIV care	PLWH have close, trusting relationships with TH	Quote 5: “[My TH] is my counsellor, my friend, and a sister … She is always there for me, and she makes sure I am on the right track. She is my teacher, family, and everything.” – 50-year-old male, PLWH Quote 6: “I had never disclosed my HIV to anyone—not even my mother. But this healer, he is more than a parent. He treats me very well. He is so close to me and special.… His care cannot be compared to health workers. They also do their best but this one is more special.” – 42-year-old female, PLWH
Factors impacting HIV clinical outcomes	TH-delivered support improved self-efficacy to remain in HIV care	Quote 7: “[The TH] made me believe and trust in myself. I am confident and ready to face whatever challenge that comes my way and continue to access my HIV services.” – 43-year-old female, PLWH Quote 8: “Before this program, I used to miss a lot my clinic appointments. Sometimes it was difficult to support myself, but that was not an issue anymore. I learnt how to plan and prepare for my next clinic appointment ahead of time and thanks to my healer who is always there to remind me, encourage me, and motivate me.” – 42-year-old female, PLWH
	TH-delivered ART readiness counseling prepared clients to reinitiate ART	Quote 9: “[The TH] took his time counseling me very well [before my appointment]. I felt I was prepared and ready to start all over my HIV treatment and care.” – 36-year-old male, PLWH
	TH effectively addressed individual barriers to ART adherence	Quote 10: “My healer, she counseled me, told me a lot about HIV, what to do, the benefits and the challenge that comes around with poor adherence. She calls me, reminds me, and sometimes checks on me where necessary to see how I take my medicine.” – 27-year-old male, PLWH Quote 11: “I monitored their ART adherence. How many tablets they were remaining, and their dates of clinic appointment. [We would] talk about their eating habits.”* –* 36-year-old female traditional healer Quote 12: “I encouraged them to involve their family members, to disclose to their spouses and others so that they keep reminding them about the time when to take their HIV medicines.” – 53-year-old male, traditional healer
	TH-delivered counseling reduced internalized stigma	Quote 13: “I had reached the worst stage that you can ever imagine but when I met this healer, it was like I was born again … I am no longer miserable. I have no more stigma. I am no longer living in self-denial. I access my HIV treatment with no challenge. I doubt I will consider stopping my HIV treatment ever again.” – 43-year-old male, PLWH
	Transportation reimbursement overcame logistic challenges to HIV care	Quote 14: “One of the main supports that was helpful was the transport which was given to us because I did not have to struggle again going to access my HIV treatment. I was motivated and encouraged because I used to be so stressed [about transportation costs].”*–* 42-year-old female, PLWH
Implementation outcomes	Program acceptability	Quote 15: “So it also helped to update clinic workers about their challenges, and any progress, of these [PLWH] clients as far as HIV treatment and management was concerned. We practically became a bridge between health care providers at the HIV clinic and the clients that we do serve.” – 56-year-old male, traditional healer Quote 16: “I found this interesting to find that traditional healers are actually helping to improve health services … I think if you are trying to address the challenges that these people face, the traditional healers will give you the first-hand information you want and the issues would be easily dealt with.” – 35-year-old male, HIV clinical liaison
	Program appropriateness	Quote 17: “As traditional healers, our [PLWH] clients find it easier to approach us because we are people who belong to the same community. We are people who have lived with them for quite a long time. They are clients whom we have served for a long time, and we do understand them very well.” – 36-year-old male, traditional healer Quote 18: “Traditional healers helped to sort out issues [to adherence] better than us in the medical sector because they know [the PLWH clients]. People believe in them more than us. They can do better in following up these people, which we can’t do. They know the challenges that people face” – 35-year-old male, HIV clinical liaison
	Program feasibility	Quote 19: “This man [my healer] is naturally gifted and talented. He is good at talking and when he says something, it is difficult to refuse or ignore whatever he says. But also, we live together, we see each other all the time and so we have a lot of time to talk and discuss.” – 43-year-old female, PLWH
	Sustainment of the program	Quote 20: “They are still on HIV care and I still support them by counselling, guiding and encouraging them never to give up and continue doing the right thing. I still monitor and follow them up. There is no getting tired because I am already connected to them and I cannot give up.” – 36-year-old male, traditional healer (12-month interview) Quote 21: “I take my HIV treatment so seriously and I have never missed taking my HIV medicines or my clinic appointments. In addition to that, I am still getting support from my healer. He is still there for me providing me with the same counselling and guidance. We see each other quite often and we discuss about my health. Nothing changed after the program ended” – 35-year-old female, PLWH (12-month interview)

Data from our *Assessment* phase indicated that logistical challenges played an important role in disengagement from care among PLWH who receive care from TH, notably geographical distance to clinics and transportation costs limiting access to care (Q1). Attending clinic appointments often meant that PLWH would have to miss income-generating activities. As such, many PLWH did not prioritize attending HIV clinic appointments. Additionally, HIV-related stigma deterred PLWH from seeking clinic-based care, thus serving as a barrier to entering and remaining in HIV care (Q2). Lack of social support for PLWH was also described as an important reason for disengagement with HIV care services (Q3). In addition, PLWH felt that they did not have sufficient rapport or trust in clinical staff to disclose adherence challenges during clinic visits (Q4). PLWH felt that their existing close, trusting relationships with TH was a foundational component for re-engaging with HIV services (Q5, Q6).

Two core elements of the intervention were identified during the *Production* phase: (1) TH facilitating rapid re-linkage to HIV care and (2) TH provision of psychosocial support using one or more of the following key elements: identifying barriers to ART adherence, healthy living, HIV status disclosure, identifying mental health symptoms.

The *Testing* phase of the study took place from September 2021 to May 2022. We enrolled 20 PLWH at 12 TH practices in the first week of September 2021 and tracked outcomes over the following 9 months. The first 20 eligible PLWH identified at the practice locations agreed to enroll. We instructed TH to aim to facilitate linkage to HIV care within 14 days of study enrollment. Client HIV status was verified through confirmatory testing at the HIV clinic per Ugandan National Guidelines [39]. The clinical liaison assisted with setting initial re-linkage to care appointments. TH provided frequent (weekly for four weeks, then monthly) psychosocial support, either in person or over the phone.

After 9 months, we contacted all PLWH participants to re-evaluate CASE adherence index, four-day ART use recall, and retention in HIV care. Baseline characteristics and 9-month results are shown in Tables 3 and 5, respectively. At baseline, the median CASE adherence score was 3 of 16 possible points; only one PLWH was adherent to ART via 4-day recall, and four were ART naive. The TH-delivered support achieved linkage and ART initiation within 14 days for all 20 PLWH, with 19/20 reporting complete ART adherence, and all being retained in care at 9 months. ART adherence improved nearly 20-fold compared with baseline (95% vs 5%).

**Table 5:** PLWH HIV outcomes after 9 months

	N (%) or median [IQR]
(Re)intiated ART within 14 days	20 (100%)
CASE adherence index	16 [15.5–16]^a^
100% ART adherence via 4-day recall	19 (95%)
Retained in HIV care	20 (100%)

^a^CASE index is out of a possible 16 points

Data from individual exit interviews illustrates how TH effectively supported re-linkage and retention in HIV care, overcoming the barriers described during the *Assessment* phase. TH-delivered social support improved self-efficacy and clients felt more willing to engage with HIV services as a result (Q7, Q8). TH also provide ART readiness counseling prior to the first relinkage appointment, which prepared clients to accept ART (re)initiation (Q9). TH achieved improved ART adherence by addressing individual barriers to adherence (Q10), including monitoring pill counts (Q11), and encouraging status disclosure and support from household members (Q12). In addition, TH-delivered counseling was described as an effective means to reduce internalized HIV-related stigma (Q13). The transportation reimbursement for the first clinical relinkage appointment provided by the study was described as an important means to offset logistical barriers faced by patients (Q14).

In May 2022, after the program had been underway for 9 months, participating TH, PLWH, and the clinic liaison completed the AIM, IAM, and FIM surveys. The adapted support program scores were 20/20 (100%) in each category, indicating the highest perceived acceptability, appropriateness, and feasibility. Acceptability measures the perception that an intervention is agreeable or satisfactory [36]. The adapted program was described as acceptable as collaborations between TH and HIV clinicians were perceived as improving the care of PLWH (Q15, Q16). Appropriateness pertains to perceived fit and relevance to the study context [36]. This TH-delivered program was perceived as appropriate because TH are well-embedded, respected members of the community and therefore effective lay supporters because they are trusted by patients (Q17, Q18). Finally, feasibility describes perceived program suitability for everyday use [36]. PLWH describe counseling as part of the existing natural “toolbox” of TH, who are already gifted and trusted counselors (Q19).

In June 2022, formal research support for the TH-delivered program ceased. Over the following three months, we assessed the degree to which the TH continued to deliver lay support as part of their routine practices through a second qualitative interview with all TH and PLWH at 12 months following the launch of the *Testing* phase. They reported remaining in contact with their clients and continuing to deliver the support in accordance with their training (Q20). Clients noted that continued support has helped them to remain adherent to ART and retained in care (Q21).

## Discussion

This mixed methods study illustrates the process of adapting an evidence-based lay supporter program for Ugandan TH and has shown that training in the adapted curriculum allowed TH to successfully support PLWH in (re)linkage to HIV care, ART adherence, and retention in care. Qualitative data demonstrate why TH-delivered lay support was particularly effective, as they provide psychosocial support, health education, and are trusted community members. By promoting communication between TH and HIV clinics, this strategy allowed TH to serve as a “bridge” between PLWH and HIV clinics, thereby improving entry and retention in the continuum of care. This approach was regarded as highly acceptable, appropriate, and feasible among those involved. Implementation data also suggest this type of program could be sustainable beyond the context of an intensely supported research program.

Approximately half of African PLWH default from care [40], with rural populations facing higher barriers to enter and remain in HIV care. TH-delivered lay support may be a strategy to improve outcomes in these specific populations, as TH are highly geographically accessible, and are less time consuming compared with clinical visits [41, 42]. Men in African countries also experience worse HIV clinical outcomes than women [43–45] as they remain outside the reach of current HIV control programs. Although this pilot study was not targeted to men, its ubiquitous success among both men and women warrants further study as to whether TH-delivered lay support may be especially impactful among men. Our findings also align with previous studies suggesting that task shifting is both cost- and clinically effective to improve outcomes in patients with chronic diseases such as diabetes, hypertension, asthma, and epilepsy [46–48]. For example, a study in South Africa indicated that task shifting using lay health workers may be used as an important resource to reduce the burden on clinical staff [49]. Consequently, TH-delivered support may be used as a strategy to engage men and rural populations, while simultaneously offloading busy HIV clinics to provide support as an adjunct to routine clinical care.

Some studies in Africa have noted that TH may contribute to delays in patients seeking HIV care and ART non-adherence [15, 17, 24, 25, 50]. Importantly, in these prior studies, TH were not trained by or formally included in HIV control programs. We have shown that with training in evidence-based strategies adapted for TH, TH can potentially improve clinical outcomes among PLWH, who have otherwise been difficult to reach via existing programs. Our rigorous use of the ADAPT-ITT stepwise process allowed for the revised program to retain its effect on improving clinical outcomes among PLWH who had defaulted from care. Our qualitative and implementation data suggest that TH-delivered lay support was welcome and impactful among PLWH who had previously defaulted from care, and has the potential to improve retention in care, and therefore HIV viral suppression. Additionally, our TH-delivered support program was unique in that it fostered collaboration and trust between TH and clinic-based clinicians, something that has been previously noted as a barrier to a successful implementation of collaborative care [18, 23].

Our study also contributes to the literature emphasizing the importance of reporting and documenting the adaptation of evidence-based interventions [51–53]. While ADAPT-ITT is one of many rigorous strategies used for adaptation [54], it emphasizes the importance of cultural context [55] and solicits stakeholder input to guide intervention adaptations to maintain fidelity to the original evidence-based program. We hope to contribute to this body of work by providing insight into methods and data used to adapt an evidence-based program for delivery by TH in rural Uganda.

### Limitations

We acknowledge that study has certain limitations. We adapted an evidence-based lay supporter program for delivery in rural Uganda for TH, an atypical group of lay providers. We acknowledge that there is heterogeneity in TH in global settings that would affect whether this program might be generalizable to implement in other contexts. However, we believe there are many shared characteristics of TH that may make this approach applicable in other settings where HIV outcomes are currently suboptimal. Second, while linkage to care, ART (re)initiation, and retention in care outcomes were confirmed by the HIV clinic, ART adherence was determined based on self-reporting. Future studies which include quantitative measures of adherence are needed to determine the impact of this program on medication adherence. While all TH were in-serviced on counseling and lay support strategies, program fidelity was not specifically evaluated as part of this pilot study, but should be specifically considered as part of scaling up this approach. Finally, we acknowledge the small sample size of participating PLWH and TH. The purpose of this study was to illustrate the adaption process and proof-of-concept. Large-scale cluster randomized trials are warranted to determine clinical effectiveness and long-term outcomes.

### Lessons learned

TH were highly motivated to learn new strategies to support their clients enter and persist in HIV care. Contextual and cultural fit are critical variables in implementation efforts, and TH are ubiquitous — though underutilized — informal providers in efforts to expand access to evidenced-based HIV care. TH are well-positioned to provide continued support as their clients are used to seeking out care at their practices, particularly in rural communities where community health workers may have greater difficulty accessing patients due to geographic distance. We note that TH-delivered interventions are distinct from community health worker-delivered interventions because patients are already independently seeking care at TH practices, and do not require recruitment strategies to attract them to these care-delivery sites. The fact that TH have a strong, existing client base strengthens core implementation domains such as intervention appropriateness and acceptability. Furthermore, formal medical clinics are sparse in rural Uganda and may lack sufficient staffing. Qualitative data revealed that TH were well-equipped to identify and address individual barriers to entering and remaining in HIV care because of pre-existing personal, trusting relationships with their clients. We note that highly individualized treatment may disrupt intervention fidelity, and therefore special attention must be paid to fidelity as this program is scaled up. Lastly, we found that clinic-based providers and TH were eager to work together and welcomed collaboration. Buy-in from participating stakeholders is imperative when designing sustainable interventions. Thus, we will continue to prioritize strengthening relationships between formal and informal providers to support intervention adoption.

## Conclusion

We guided the adaption of an evidence-based lay support intervention for PLWH following the ADAPT-ITT framework to create a training program for TH in rural Uganda. After receiving this adapted curriculum, TH successfully facilitated re-linkage to HIV care and supported ART adherence in the short-term among PLWH who had defaulted from HIV care. Future studies are needed to assess scale-up and clinical effectiveness in the long-term.

## Data Availability

The datasets used and/or analyzed during the current study are available from the corresponding author on reasonable request.
